# Efficacy of low-dose radiotherapy (2 Gy × 2) in the treatment of marginal zone and mucosa-associated lymphoid tissue lymphomas

**DOI:** 10.1259/bjr.20210012

**Published:** 2021-06-16

**Authors:** Marzia Cerrato, Erika Orlandi, Angelisa Vella, Sara Bartoncini, Giuseppe C Iorio, Diego Bongiovanni, Francesco Capriotti, Carola Boccomini, Francesco Vassallo, Chiara Cavallin, Viola De Luca, Francesca R Giglioli, Umberto Ricardi, Mario Levis

**Affiliations:** 1 Department of Oncology, University of Torino, Torino, Italy; 2 Hematology Unit, Azienda Ospedaliero Universitaria Città della Salute e della Scienza di Torino, Torino, Italy; 3 Medical Physics Unit, A.O.U. Città della Salute e della Scienza, Torino, Italy

## Abstract

**Objectives:**

To investigate the efficacy of a schedule of low-dose radiotherapy (LDRT) with 4 Gy (2 Gy x 2) in a cohort of unselected MALT or MZL patients.

**Methods:**

We retrospectively collected all patients receiving LDRT, either for cure or palliation, for a stage I–IV histologically proven MALT or MZL between 2016 and 2020. Response to LDRT was evaluated with the Lugano criteria. Local control (LC), distant relapse-free survival (DRFS), progression-free survival (PFS) and overall survival (OS) were stratified for treatment intent (curative *vs* palliative) and estimated by the Kaplan-Meier product-limit.

**Results:**

Among 45 consecutively enrolled patients with a median age of 68 years (range 22–86), 26 (58%) were female. Thirty-one patients (69%) with a stage I–II disease received LDRT as first line therapy and with a curative intent. Overall response rate was 93%, with no significant difference among curative and palliative intent. With a median follow-up of 18 months, LC, DRFS, PFS and OS at 2 years were 93, 92, 76 and 91%, respectively, in the overall population. Patients receiving curative LDRT had a better PFS at 2 years (85% *vs* 54%, *p* < 0.01) compared to patients receiving palliative treatment. LDRT was well tolerated in all patients, without any significant acute or chronic side-effect.

**Conclusions:**

LDRT is effective and well tolerated in patients affected with MALT or nodal MZL, achieving high response rates and durable remission at 2 years.

**Advances in knowledge:**

This study shows the efficacy of LDRT in the treatment of MALT and MZL.

## Introduction

Marginal zone B-cell lymphomas (MZLs) represent approximately 5–15% of all non-Hodgkin lymphomas (NHL).^1^ MZLs are classified as indolent lymphomas and the most frequent subtype is the mucosa-associated lymphoid tissue (MALT) lymphoma, which can involve many organs and extranodal sites. Localized forms are rare for nodal MZLs, while MALT lymphomas present with a single extranodal involvement in more than 70% of cases, making local treatment the preferred initial approach.^2^ In both nodal and extranodal early stage MZLs, the curative role of involved-site radiotherapy (RT) is well established, with radiation dose ranging 24–30 Gy.^2–4^ In advanced stage disease, RT can still be used for palliation of symptoms or for cytoreduction.^5–7^ Historically, conventional-dose RT provides excellent local control (LC) rates, frequently approaching 95%.^8^

Given the high radiosensitivity of indolent lymphomas, there has been a recent trend in treating these histotypes with a dose of radiation, reduced to as low as 4 Gy delivered in two consecutive fractions of 2 Gy. This alternative “Low-dose” (LDRT) fractionation schedule proved to be effective for palliation in advanced stage and relapsed disease.^5–7,9–12^ In the curative setting, LDRT was inferior to the 24 Gy regimen in the treatment of early-stage indolent lymphomas in the Phase III randomized FORT trial.^4^ The long-term follow-up results were recently published, stating that 24 Gy should be regarded as the standard of care for indolent lymphomas, while 4 Gy represents a valuable alternative for palliative treatment.^13^ Nevertheless, indolent subtypes seem to have a distinct response to LDRT. In fact, MALT and nodal MZLs achieved high response rates with 4 Gy, often resulting in complete and durable remission, in limited retrospective case series.^14–18^ However, all these reports included few patients, with a single site of MALT (mainly orbital/ocular-adnexa) involvement.

In this study, we aimed to evaluate the efficacy of LDRT (4 Gy in two fractions) and to report the clinical outcomes of a cohort of unselected and consecutive nodal MZLs and extranodal MALT lymphoma patients treated at our institution.

## Methods and materials

### Study design and sample size

We retrospectively collected all patients diagnosed with a MALT or nodal MZL that accepted to receive LDRT with either curative or palliative intent as an alternative to the standard dose schedule of 24 Gy in 12 fractions. All patients were treated at the Department of Oncology of the University of Torino between January 2016, when the authors adopted LDRT fractionation regimen for the first time, and June 2020.

Patients affected with a histologically proven early- or advanced-stage MALT/MZL, according to the World Health Organization classification, were eligible, regardless of the location and of the tumour burden. All patients were staged in accordance with the international guidelines.^19^ Bone marrow biopsy was routinely executed and was mandatory for stage I and II patients. Radiographic staging and treatment revaluations varied on the basis of the anatomical presentation and included total body computed tomography, magnetic resonance, positron emission tomography, ultrasound and mammography. All clinical, treatment related and follow-up details were retrieved from the digitalized patients’ charts. The study was authorized by our Institutional Review Board and was conducted in respect with the Italian law.

### Treatments details

A personalized radiation treatment was planned for each patient and the target volumes were delineated on the basis of both the anatomic site and the extent of disease, as described by the ILROG guidelines for nodal and extranodal non-Hodgkin lymphomas.^20,21^ Therefore, regional uninvolved lymph nodes were not included in the target of radiation. LDRT was delivered with 4 Gy in two fractions of 2 Gy on two consecutive days. For most patients, treatment was planned with a 3D conformal photon-based technique (93%). In the case of superficial lesions, limited to the skin (three patients), treatment was delivered using electron beams (6–10 MeV). Details on previous local or systemic treatments for the same lymphoma were collected.

### Response assessment and follow-up

All patients were regularly followed up with clinical visits and radiological studies after LDRT treatment. Initial response was assessed 2–3 months after LDRT. Response rates were classified as complete response (CR), partial response (PR), stable disease (SD) and progressive disease (PD). CR and PR were combined to define the overall response. Response was based on clinical assessment (primarily for superficial and skin lesions) and on radiographic studies of restaging, in respect of the Lugano classification.^22^ Therefore, CR was defined as a complete macroscopic resolution of the disease and PR as a reduction of the tumour burden >50% compared to baseline. No tumour volume reduction identified SD, while an increase in tumour volume was scored as PD.

All patients then received follow-up visits every 4–6 months and the repetition of radiographic exams was left to the discretion of the treating physician. Progression and relapse were classified as any measurable or visible increase of known sites or the appearance of new sites of disease.

### Statistical considerations

The primary endpoint of this study was LC, with local relapse defined as any event occurring within the radiation field. Secondary endpoints were distant relapse free survival (DRFS), progression free survival (PFS) and overall survival (OS).

Distant relapse was defined as any event occurring outside the LDRT field, while PFS accounted for any event among local relapse, distant relapse and death.

Time to event was calculated from the end of LDRT for all clinical endpoints. Patients alive and without disease relapse at the last assessment have been censored at the date of the last follow-up.

Time-to-event functions have been estimated with the Kaplan-Meier product-limit. Hazard Ratios (HRs) have been calculated using the Cox proportional-hazards model. Factors with *p* < 0.05 were considered statistically signiﬁcant. All statistical analyses were performed with SPSS Statistics version 26.0 (SPSS Inc., Chicago, Illinois, USA).

## Results

### Patient characteristics

We reviewed 45 consecutive patients affected with a histologically proven MALT or nodal MZL. Forty-one participants (91%) had a MALT lymphoma, while four patients (9%) were diagnosed with a nodal MZL. Only one patient had a bulky lesion (inguinal lymph node with maximum diameter of 6 cm) at the time of LDRT. The site of involvement was orbital in 16 patients (35%), head and neck region in 12 patients (27%), while the remaining 17 subjects (38%) had a disease presentation in other sites (skin, subcutaneous tissue, lymph nodes, breast, lung, para-cardiac, spleen, liver, kidney, mesorectal fascia and spinal canal). Most patients were female (58%) and the median age at time of LDRT was 68 years (range 22–86). Thirty-seven patients (82%) had localized disease (stage I–II according to the Ann Arbor staging system) at the time of enrolment. Patient and tumour characteristics are detailed in Table 1.

**Table 1.** Patients characteristics

Number of patients		45
Median age (range)		68 (22–86)
Gender, no. (%)	Male	19 (42,2%)
	Female	26 (57,8%)
Histhology, no. (%)	MZL, MALT type	45 (100%)
Lesion site, no. (%)	Ocular- adnexa	16 (35,5%)
	Head and Neck	12 (26,7%)
	Others^*a*^	17 (37,8%)
Stage no. (%)	I	36 (80%)
	II	1 (2,2%)
	III	1 (2,2%)
	IV	7 (15,6%)
Bulky^*b*^ lesions, no. (%)	Yes	1 (2,2%)
	No	44 (97,8%)
Diagnostic Imaging, no. (%)	Mx	1 (2,2%)
	US	8 (17,8%)
	CT	39 (86,7%)
	MRI	22 (48,9%)
	PET	10 (22,2%)

CT, Computed tomography; MALT, mucosa-associated lymphoid tissue; MRI, Magnetic resonance imaging; MZL, Marginal zone lymphoma; Mx, Mammography; PET, Positron emission tomography; US, Ultrasound imaging.

a Others = breast+kidney, (1) lung, (1) thyroid, (1) liver, (1) dura mater, (1) spleen, (2) skin, (3) subcutis, (2) para-bladder, (1) para-cardiac, (1) lymph nodes (4).

b Bulky = maximum diameter > 5 cm.

Most patients received LDRT as first-line therapy and with curative intent (31 patients, 69%). Remaining patients (31%) received LDRT for palliation of a relapsed disease or for a MALT/MZL diagnosed in advanced stage. Previous local or systemic treatments were administrated in 10 cases. Further treatment details are listed in Table 2.

**Table 2.** Treatments characteristics

Number of treated lesions, no. (%)		45
Previous treatment, no. (%)	Prior Surgery	4 (8,9%)
	Prior Chemoterapy	1 (2,2%)
	Prior Radiotherapy	1 (2,2%)
	Prior combined therapy^*a*^	4 (8,9%)
Radiation modality, no. (%)	Photons (3D-CRT)	42 (93,3%)
	Electrons (6–10 MeV)	3 (6,7%)
Treatment schedules, no. (%)	4 Gy/2 fr	45 (100%)
Reason for Radiotherapy, no. (%)	Curative intent	31 (68,9%)
	Palliative intent	14 (31,1%)

a Combined therapy = Rituximab+Chemiotherapy (3), Rituximab + Surgery (1).

**Figure 1.**
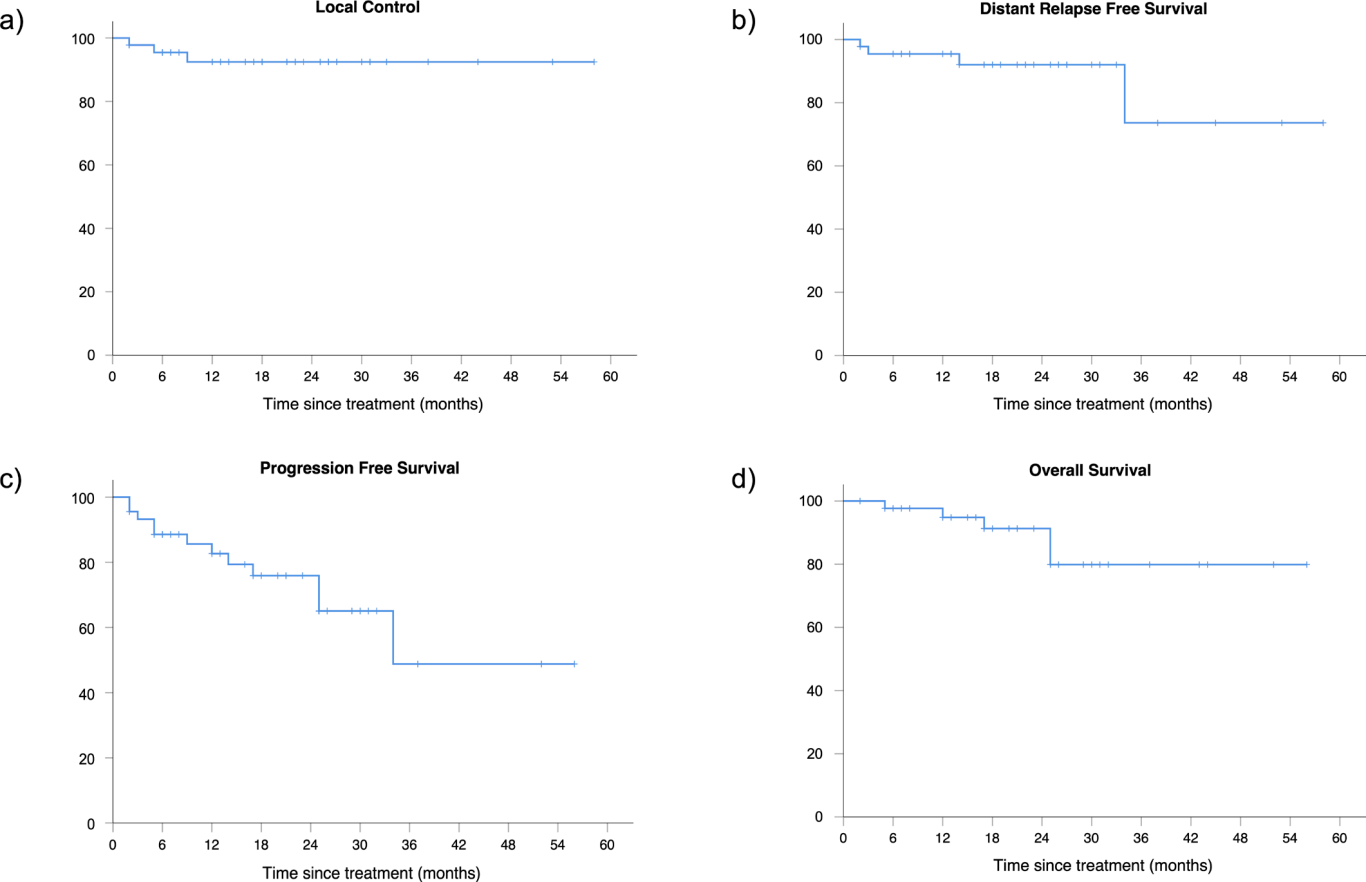
Local control (**a**), distant relapse free survival (**b**), progression free survival (**c**) and overall survival (**d**) of the entire cohort.

**Figure 2.**
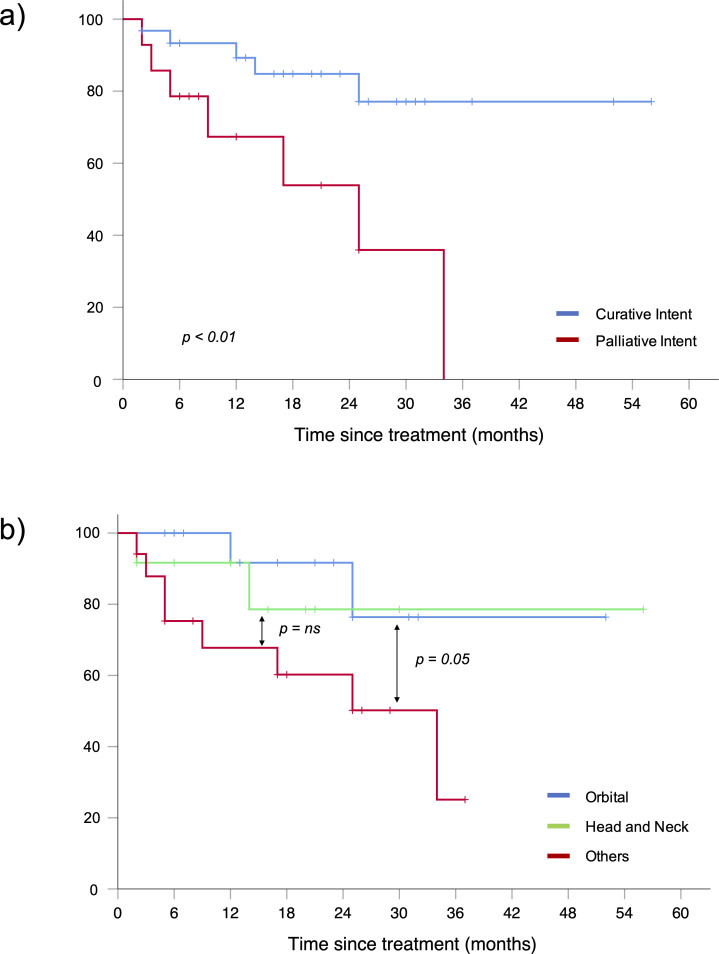
Progression-free survival stratified for treatment intent (**a**) and for the site of disease involvement (**b**).

Overall response rate (ORR) after LDRT was 93% (CR 51%, PR 42%). In patients treated with curative intent ORR was slightly superior, but without reaching a statistical significance, compared to patients receiving palliative treatment (97% *vs* 86%, respectively). Only three patients had a SD after LDRT and no PD was recorded. With a median follow-up time of 18 months (range 3–58), 2 years LC was 93%. Of the three patients (7%) with local in-field relapse (from 2 to 9 months after RT), one showed a PR, one achieved a CR and one was in SD at the first reassessment after LDRT. Each patient was re-treated with a radiation dose of 20 Gy in 10 fractions. Two of them achieved CR and 1 PR and they are all in remission at the last follow-up. DRFS, PFS and OS were respectively 92, 76 and 91% at 2 years. (Figure 1). After stratification, we found that patients receiving LDRT with curative intent had a better PFS at 2 years compared to patients undergoing palliative treatment (85% *vs* 54%, respectively, *p* < 0.01) (Figure 2a). Treatment intent did not impact in the same measure on OS (96% *vs* 80% for curative and palliative LDRT, respectively, *p* = 0.06) (Table 3). During follow-up, five patients died. One patient died of pneumonia related to the COVID-19 pandemic and four patients died for unknown causes unrelated to the lymphoma, as no one had a local or a systemic relapse during the follow-up time.

**Table 3.** Treatment outcomes to low-dose radiotherapy (2 Gy x 2)

	ORR	CR	PR	Local relapse^a^	Distant relapse	2yPFS	2yOS
All patients (*n* = 45)							
% of patients	93	51	42	7	9	76	91
Curative intent (*n* = 31)							
% of patients	97	58	39	6	3	85	96
Palliative intent (*n* = 14)							
% of patients	86	36	50	7	21	54	80

CR, Complete response; ORR, Overall response rate; OS, Overall survival; PFS, Progression free survival; PR, Partial response.

a In field progression.

After stratification for the site of disease, we identified three subgroups: orbital (16 patients), head and neck (12 patients) and other sites (17 patients). We observed a better PFS at 2 years for the orbital subgroup compared to other sites (91% *vs* 60%, *p* = 0.05) (Figure 2b).

**Figure 3.**
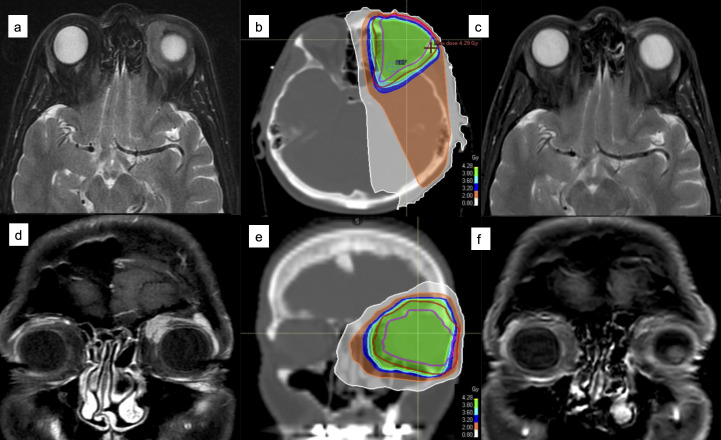
A 55-year-old lady diagnosed with stage I ocular MALT (**a, d**) was treated with LDRT (**b, e**) in a curative setting. She obtained CR at the first MRI revaluation (**c, f**) and is still well being and in complete remission 1 year after radiotherapy.

**Figure 4.**
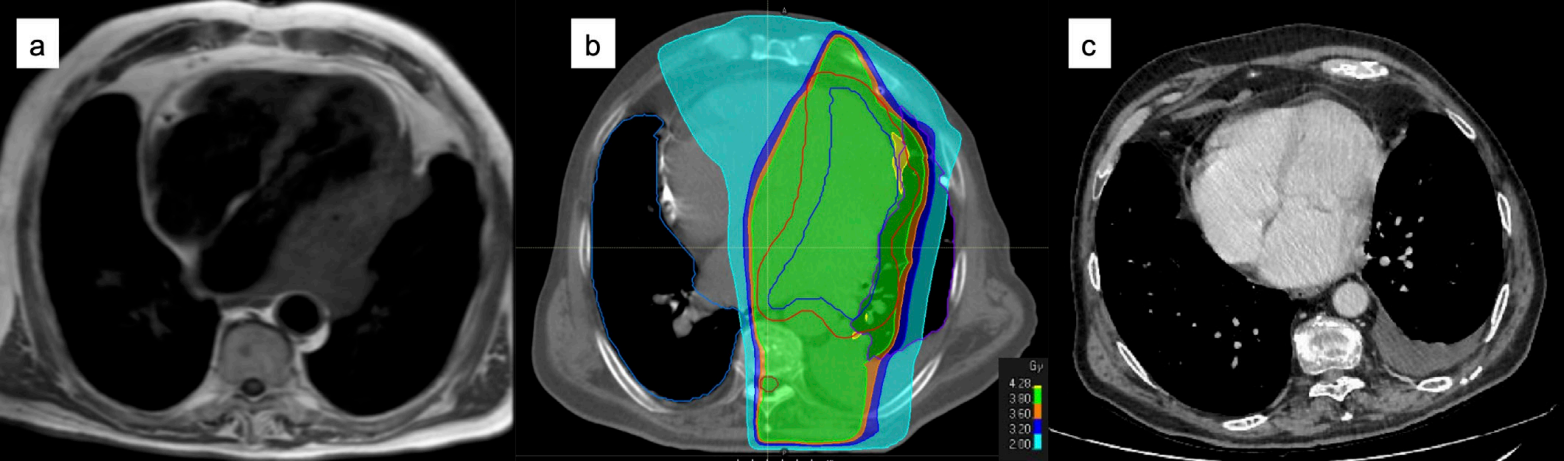
A 69-year-old male was treated with RT (24 Gy/12 fr) in 2014 for an ocular MALT. In 2017, he complained dyspnoea and asthenia. TC and cardiac MRI showed a para-cardiac lesion (**a**) that was biopsied, confirming a MALT histology also in this site. He underwent LDRT with a palliative intent (**b**) and a persistent CR was obtained (**c**). Unfortunately, in 2020 he experienced a systemic progression of disease and was treated again with Rituximab in association with chemotherapy. The patient is currently alive and in good PR.

Figures 3 and 4 show two examples of CR to LDRT, respectively in the curative and in the palliative setting.

LDRT was well tolerated in our population, without any significant acute or late side-effect. In particular, all patients with orbital localization had a valuable clinical response to LDRT, with relief of onset symptoms in few weeks by the end of treatment and no evidence of the side-effects usually detected after standard-dose RT.

## Discussion

Radiotherapy is an effective treatment strategy for patients with MALT and nodal MZLs. In the curative setting, RT alone with a dose of 24 Gy is the standard of treatment in stage I–II disease. Standard-dose RT provides excellent local control rates (>95%) and durable PFS in more than 70% of the treated patients.^8^ Given the high radiosensitivity and the usually favourable outcome, indolent NHL are increasingly treated with LDRT, with promising results. The first data on the efficacy of LDRT dates back to 1994, when Ganem and colleagues reported high response rates (ORR 89%) to 4 Gy schedule in a cohort of advanced-stage indolent lymphomas treated for palliation.^9^ Haas et al^7^ tested LDRT in 109 patients with various indolent subtypes (only 9 MALT/MZL included), achieving an ORR of 92% (CR rate was 61%, and PR rate was 31%). These results were then confirmed in several retrospective studies^10–12^ with ORR ranging from 75 to 95%. The wide range of response rates reflects the heterogeneity in patient cohorts, both in terms of histology, disease stage and affected areas. In light of these promising results, United Kingdom Cancer Research has investigated the efficacy of LDRT, compared to the standard dose of 24 Gy, in a prospective randomized, unblinded, Phase III non-inferiority study (“FORT” study).^4,13^ The study included 548 patients treated with either curative or palliative intent, showing higher response rates with 24 Gy (ORR: 91% *vs* 81%; CR: 68% *vs* 49%, *p* < 0.001) and longer time to local progression (HR 3.42, *p* < 0.001). Therefore, the FORT study failed to prove the non-inferiority of 4 Gy in indolent NHL. These results supported the use of 4 Gy in indolent NHL, as a valuable alternative to the standard regimen of 24 Gy, only for palliation, or in patients with poor performance status. Nevertheless, all mentioned studies, including the FORT trial, mainly focused on follicular lymphoma and enrolled a limited number of MALT/MZL patients (10–20%), leaving the question on the effect of LDRT in non-follicular indolent lymphomas unanswered. In fact, the FORT trial patients with MALT and MZL had better response rates to LDRT compared to other indolent histologic types, as shown by a similar ORR for 24 Gy and 4 Gy (92% *vs* 87%, respectively, *p* = 0.71).^4^ To date, only few studies have investigated LDRT in MALT and MZL, all addressing a specific site of disease within limited cohorts of patients..^14–18^

To our knowledge, we present herein the largest retrospective study of unselected and consecutive nodal and extranodal marginal zone lymphomas treated with LDRT at a single institution. In a population of 45 patients, we observed an ORR of 93% (CR: 51%, PR: 42%) and a 2 year LC of 93% with 4 Gy. Our results are consistent with those presented by the FORT trial for 24 Gy in MALT/MZL. After stratification for the treatment intent, we identified 31 patients who received LDRT in the curative setting: in these, PFS and OS at 2 years were 85 and 96%, respectively. In particular, the PFS rate at 2 years was similar to that achieved by the group from the Memorial Sloan Kettering Cancer Center with 24 Gy in the largest report (244 patients) available to date for stage I–II MALT lymphomas.^8^ Despite achieving similar LC rates, in patients treated for palliation we observed an expectedly lower (54%) PFS at 2 years compared to patients receiving curative treatment.

Our study reinforces, on a larger and heterogeneous cohort of MALT and MZL, the results of previous reports focused on limited and highly selected cohorts of patients. In 2012, Girinsky and colleagues first published a paper on 10 pulmonary MALT treated with LDRT, reporting a 5 year PFS of 87% and OS of 100%.^14^ Two successive studies have demonstrated the efficacy of 4 Gy for orbital MALT lymphomas, with an ORR ranging 95–100% and durable LC in >90% of patients.^15,16^

Also, orbital lymphomas benefit the most from the reduction of radiation doses to as low as 4 Gy for a concomitant decrease of treatment-related toxicity, which may include both immediate (eye lid irritation, conjunctivitis) and late complications (cataract formation, dry eye syndrome and more rarely macular degeneration)^23,24^ with higher doses of RT (>30 Gy). Our study included 16 patients with an orbital MALT and none experienced any significant acute or chronic side-effect.

More recently, few reports have investigated the role of 4 Gy in cutaneous,^17^ head and neck,^25^ salivary gland^26^ and breast^18^ localizations of MALT lymphomas, all demonstrating the efficacy of LDRT in terms of LC. We observed the same high LC rates for all different sites of MALT or nodal MZL, but with a distinction in terms of PFS. Indeed, orbital MALT had a better PFS at 2 years compared to other “non-head and neck” sites (91% *vs* 60%, *p* = 0.05) in our study. The reasons for a better outcome for patients with orbital lesions could be the presence of anatomical boundaries in the orbit and the lower tumour burden compared to other sites. In addition, patients with orbital MALT often have visible disease or present with symptoms such as mass effect or decreased visual acuity that may prompt earlier diagnosis and treatment.^15^ However, our observation is limited by the modest number of patients with MALT lymphomas in other sites and by the short follow-up, requiring a future confirmation.

In our study, only three patients experienced a local relapse in the following MALT sites: skin, subcutaneous tissue and nasopharynx. In these cases, patients were offered a retreatment consisting of additional 20 Gy in 10 fractions, and all achieved a complete and durable remission. Two ongoing prospective studies led by the MD Anderson Cancer Center are testing the same treatment option for orbital (NCT02494700) and gastric (NCT03680586) MALT lymphomas. This strategy seems very promising to us. Indeed, it allows to spare undue treatment toxicity and to increase patient compliance for the limited treatment time, without compromising the final outcome for the possibility to offer retreatment in case of failure or relapse after LDRT. In other studies, the relapsing site was successfully retreated with a new cycle of LDRT,^15,27^ with promising results. Whatever the strategy selected, it is important to underline that treatment with LDRT does not hamper further retreatment with radiation therapy, if necessary.

The main limitations of our study are the retrospective nature and the short follow-up. Indeed, the median follow-up time of 18 months might be not sufficient to detect local or distant relapses, which can occur even several years after treatment.^2,28,29^ Furthermore, our study does not include patients with gastric involvement, which is the most common for MALT lymphomas.^19^ This is because the standard follow-up requires repeated invasive procedures, such as esophagogastroduodenoscopy (EGD) and invasive biopsies. Future prospective studies on larger cohorts including all sites of MALT and nodal MZL are warranted to confirm the role of LDRT in this setting.

## Conclusions

Our results suggest that LDRT is effective and well tolerated in patients affected with MALT or nodal MZL, achieving high response rates and local control at 2 years. In patients treated with curative intent, PFS rate at 2 years is similar to that achieved with the standard dose of 24 Gy. Longer follow-up time is needed to confirm a durable local and systemic remission for LDRT.
